# Derivation and validation of a lipid-covered prognostic model for mature T-cell lymphomas

**DOI:** 10.1186/s12935-021-02042-3

**Published:** 2021-07-05

**Authors:** Tiange Lu, Lei Shi, Guanggang Shi, Yiqing Cai, Shunfeng Hu, Jiarui Liu, Shuai Ren, Xiangxiang Zhou, Xin Wang

**Affiliations:** 1 Department of Hematology, Shandong Provincial Hospital, Cheeloo College of Medicine, Shandong University, No. 324, Jingwu Road, Jinan, 250021 Shandong China; 2 Department of Hematology, Shandong Provincial Hospital Affiliated to Shandong First Medical University, Jinan, 250021 Shandong China; 3 School of Medicine, Shandong University, Jinan, 250012 Shandong China; 4 Department of Otolaryngology-Head and Neck Surgery, Shandong Provincial Hospital Affiliated to Shandong First Medical University, Jinan, 250021 Shandong China; 5 Shandong Provincial Engineering Research Center of Lymphoma, Jinan, 250021 Shandong China; 6 Branch of National Clinical Research Center for Hematologic Diseases, Jinan, 250021 Shandong China; 7 National Clinical Research Center for Hematologic Diseases, The First Affiliated Hospital of Soochow University, Suzhou, 251006 China

**Keywords:** Dyslipidemia, Prognosis, EnBC score, Mature T-cell lymphomas

## Abstract

**Background:**

Mature T-cell lymphomas (MTCLs), a group of diseases with high aggressiveness and vulnerable prognosis, lack for the accurate prognostic stratification systems at present. Novel prognostic markers and models are urgently demanded. Aberrant lipid metabolism is closely related to the tumor progression but its prognostic significance in MTCLs remains unexplored. This study aims to investigate the relationship between dysregulated lipid metabolism and survival prognosis of MTCLs and establish a novel and well-performed prognostic scoring system for MTCL patients.

**Methods:**

A total of 173 treatment-naive patients were enrolled in this study. Univariate and multivariate Cox regression analysis were performed to assess the prognostic significance of serum lipid profiles and screen out independent prognostic factors, which constituted a novel prognostic model for MTCLs. The performance of the novel model was assessed in the training and validation cohort, respectively, by examining its calibration, discrimination and clinical utility.

**Results:**

Among the 173 included patients, 115 patients (01/2006–12/2016) constituted the training cohort and 58 patients (01/2017–06/2020) formed the validation cohort. Univariate analysis revealed declined total cholesterol (TC, P = 0.000), high-density lipoprotein cholesterol (HDL-C, P = 0.000) and increased triglycerides (TG, P = 0.000) correlated to inferior survival outcomes. Multivariate analysis revealed extranodal involved sites ≥ 2 (hazard ratio [HR]: 2.439; P = 0.036), β2-MG ≥ 3 mg/L (HR: 4.165; P = 0.003) and TC < 3.58 mmol/L (HR: 3.338; P = 0.000) were independent predictors. Subsequently, a novel prognostic model, EnBC score, was constructed with these three factors. Harrell’s C-index of the model in the training and validation cohort was 0.840 (95% CI 0.810–0.870) and 0.882 (95% CI 0.822–0.942), respectively, with well-fitted calibration curves. The model divided patients into four risk groups with distinct OS [median OS: not available (NA) vs. NA vs. 14.0 vs. 4.0 months, P < 0.0001] and PFS (median PFS: 84.0 vs. 19.0 vs. 8.0 vs. 1.5 months, P < 0.0001). Time-dependent receiver operating characteristic curve and decision curve analysis  further revealed that EnBC score provided higher diagnostic capacity and clinical benefit, compared with International Prognostic Index (IPI).

**Conclusion:**

Firstly, abnormal serum lipid metabolism was demonstrated significantly related to the survival of MTCL patients. Furthermore, a lipid-covered prognostic scoring system was established and performed well in stratifying patients with MTCLs.

**Supplementary Information:**

The online version contains supplementary material available at 10.1186/s12935-021-02042-3.

## Background

Mature T-cell lymphomas (MTCLs) comprise a large spectrum of highly aggressive and heterogeneous lymphatic diseases originating from post-thymic T-lymphocytes and NK-cells [1–3]. This group of diseases can be roughly divided into 4 categories, including: disseminated or leukemic disease, nodal disease, extranodal disease and cutaneous disease [4]. Compared with B-cell lymphoma counterparts [5], T-cell lineage lymphomas are characterized by lower morbidity, poorer prognosis and higher mortality [6]. The heterogeneity of genetic background, tumor burden and clinicopathological characteristics leads to the variant therapeutic responses and survival outcomes [7]. The precise prognosis stratification and individualized therapy are expected to extend MTCL patient’s survival.

However, the prognostic markers and risk stratification systems for MTCLs are badly deficient in contrast with B-cell lymphomas. The International Prognostic Index (IPI) has been revealed a number of defects in stratifying MTCLs [4] since the patients even in the low risk category (IPI 0 or 1) still exhibited severely-damaged outcomes and the curves seen for MTCL patients essentially identified 2 risk categories (IPI ≤ 1 and IPI ≥ 2) with limited separation [4]. In addition, the IPI was revealed unfitted for stratifying some certain MTCL subtypes, such as adult T-cell leukemia/lymphoma (ATLL), enteropathy-associated T-cell lymphoma (EATL), hepatosplenic TCL and extranodal NK/T-cell lymphoma (ENKTL) [8]. Consequently, there is an urgent need to seek for novel markers and develop a universal risk scoring system for patients with MTCLs.

Lipids, as the key components of cell energy metabolism, biological membrane synthesis and signal transduction, play indispensable roles in the occurrence and progression of tumors [9]. With the ripeness of lipidomic technologies, lipid metabolism reprogramming has been regarded as an emerging hallmark of tumor [10]. An increasing body of evidences indicate that lipid metabolism disorder closely involves in the tumor progression but exerts contradictory influence on the prognoses of patients in variant cancers [11]. It has been reported that disrupted cholesterol homeostasis is associated with the poor prognosis and high serum levels of cholesterol increase anti-tumor functions of NK-cells in hepatocellular carcinoma (HCC) [12, 13]. Differently, another study found that high cholesterol levels were positively correlated with rectal cancer risk [14].

Nevertheless, the association between the aberrant lipid metabolism and the prognosis of MTCLs remains undiscovered. In this study, we investigate the prognostic significance of lipid metabolism abnormality in MTCL patients. Furthermore, we seek to establish an effective prognostic scoring system for MTCLs through synthetically analyzing patients’ clinicopathologic features and laboratory examinations. The predictive performance of the new model would be verified from multiple dimensions and tested in an independent validation cohort. Its superiority would be demonstrated by comparison with the existing prognostic scoring system.

## Methods

### Study population

We retrospectively analyzed newly diagnosed MTCL patients hospitalized at Shandong Provincial Hospital from January 2006 to June 2020. Patients treated from January 2006 to December 2016 formed the training cohort and patients from January 2017 to June 2020 made up the validation cohort. Two cohorts followed the same inclusion and exclusion criteria. The inclusion criteria contain: (1) pathologically confirmed diagnosis of MTCLs based on the WHO 2016 Classifications of Tumors of Hematopoietic and Lymphoid Tissues [7], (2) treatment-naïve. Exclusion criteria include: (1) with incomplete clinical data and follow-up information, (2) with previous malignancies or major diseases.

### Data collection

The baseline data, such as gender, age, extranodal sites, bone marrow involvement, Eastern Cooperative Oncology Group (ECOG) score, Ann Arbor stage, IPI and B symptoms were gathered. Laboratory examinations, including serum lactate dehydrogenase (LDH), β2-microglobulin (β2-MG), total cholesterol (TC), triglycerides (TG), high-density lipoprotein cholesterol (HDL-C) and low-density lipoprotein cholesterol (LDL-C) were collected.

### Treatment

Broadly speaking, therapy schemes comprised chemotherapy (CT) alone, radiotherapy (RT) alone, chemoradiotherapy (CRT), chemotherapy combined with histone deaceylase inhibitor (Chidamide) and chemotherapy joint with hematopoietic stem cell transplantation (HSCT).

For patients with ENKTL, treatment schedules varied according to different stages. Patients in localized stage were treated with RT alone or concurrent chemoradiotherapy (CCRT), such as RT-DeVIC (RT with dexamethasone, etoposide, ifosfamide, and carboplatin) and CCRT-VIPD (CCRT with etoposide, ifosfamide, cisplatin and dexamethasone). Whether extended involved-site intensity-modulated radiotherapy (IMRT) or three-dimensional conformal radiotherapy both reached ≥ 50 Gy dose. Advanced ENKTL patients mainly received pegaspargase-covered or gemcitabine-covered CT, including SMILE (dexamethasone, methotrexate, ifosfamide, pegaspargase, and etoposide), GELOX (gemcitabine, pegaspargase, and oxaliplatin) and MESA (methotrexate, etoposide, dexamethasone and pegaspargase).

Patients with MTCLs other than ENKTL were mostly treated with CT alone. CT regimens covered CHOPE [Cyclophosphamide, doxorubicin, vincristine, prednisone (CHOP) and etoposide], hyperCVAD (cyclophosphamide, vincristine, doxorubicin and dexamethasone for A protocol; high-dose methotrexate plus cytarabine for B protocol), BACOP (CHOP and bleomycin), COMP (cyclophosphamide, vincristine, methotrexate and prednisone) and DICE (dexamethasone, ifosfamide, cisplatin and etoposide).

### Follow-up

The follow-up data was obtained by inpatient medical records, outpatient check-ups or phone interviews. All data, including basic and serological information, treatments and outcomes were prospectively collected and retrospectively analyzed. The primary observation endpoint was overall survival (OS), followed by progression-free survival (PFS). OS was defined as the period from the date of diagnosis to the date of last follow-up or all-cause death. PFS was calculated as the interval from diagnosis to the first disease progression or last follow-up.

### Statistical analyses

Continuous variables that did not fit the normal distribution were reported as medians [interquartile range (IQR)] and compared using the Mann–Whitney U test. Normally distributed variables, reported as mean ± (standard deviation), were compared using the Student *t* test. Categorical data, presented as frequency (%), were compared using the Chi-squared test or Fisher’s exact test. The dichotomous cutoff values of serum lipid compositions’ concentrations were determined by receiver operating characteristic (ROC) curves according to the maximal associated J statistic (Youden’s index). Univariate and multivariate analysis of the training cohort were conducted to detect independent predictors. Variables significantly associated with survival in the univariate analysis (P < 0.05) were brought into multivariate regression model and the independent predictors derived from multivariate analysis constituted the novel model, whose point was assigned according to the regression coefficients (B). Calibration curves were plotted to estimate the predictive accuracy. OS and PFS survival curves were constructed using the Kaplan–Meier method and compared using the log-rank test. Time-dependent ROC curve analysis, Brier score and decision curve analysis (DCA) were performed to determine the superior model. Time-dependent areas under ROC curve (AUCs) and Brier score were measured dynamically. Decision curve analysis was conducted to examine which model possesses the better clinical net benefit. P < 0.05 was considered statistically significant and all tests were two-tailed. Statistical analyses were executed by MedCalc 15.2.2.0, SPSS 25.0 (SPSS, Chicago, IL, USA) and R program (version 3.6.2; R Foundation for Statistical Computing, Vienna, Austria). Several packages were used in the R environment, including “survival”, “survminer”, “CsChange”, “stdca”, “Time-ROC” and “rms”.

## Results

### Clinical features, treatment and survival

**Fig. 1:**
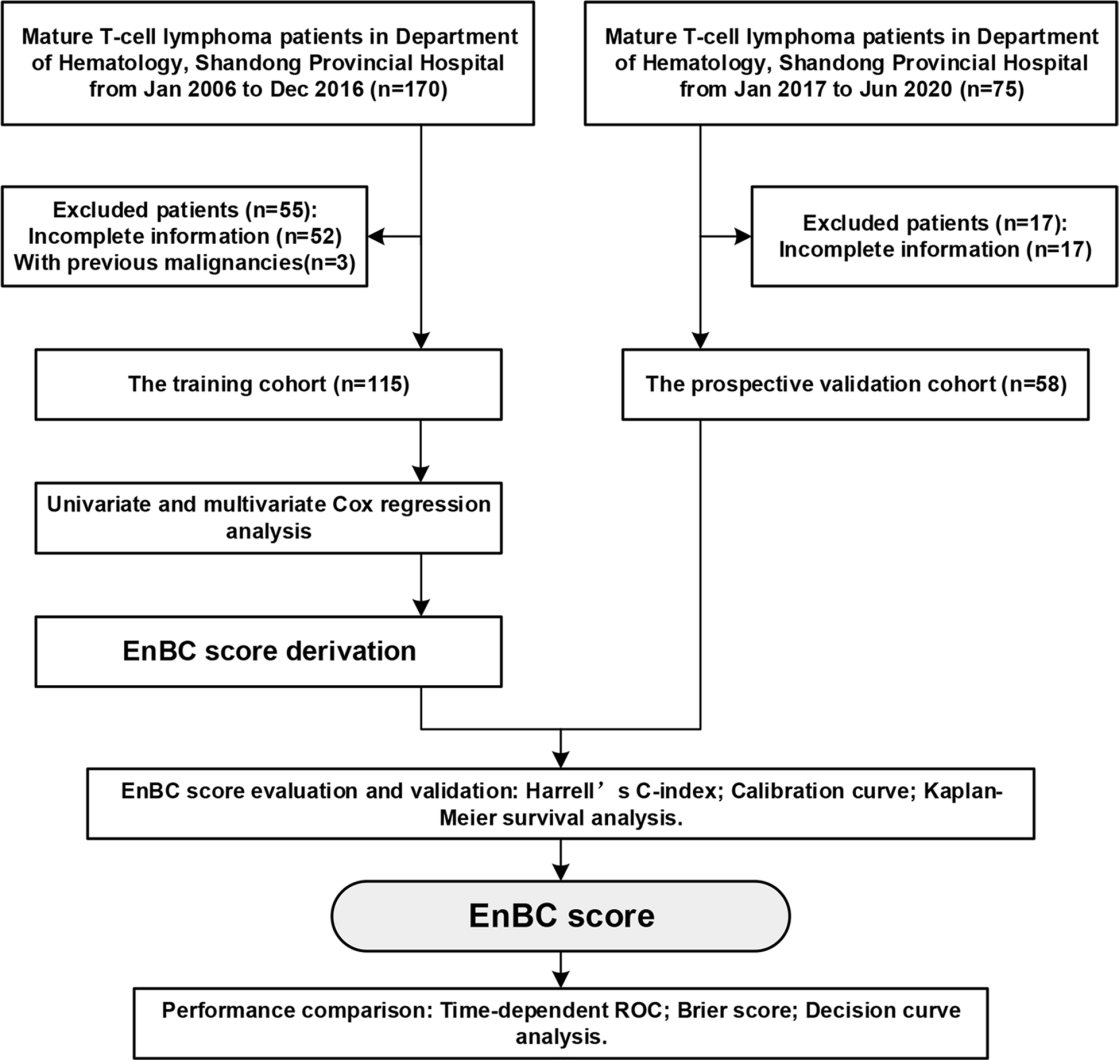
Flow chart of cohort constitution and model development

The flow chart (Fig. 1) presents the process of the study, including the constitution of the training and validation cohorts, the derivation and validation of the novel model and the comparison between the model and IPI. A total of 173 patients were included in the study, 115 patients in the training cohort and 58 patients in the validation cohort. The baseline clinical and biologic characteristics of patients in the training and validation cohort were exhibited in Table 1. Most characteristics between two cohorts were similar. The median onset ages of the training and validation cohort were 54 and 52 years old (P = 0.611) and male accounted for the majority, 69.6% and 67.2% (P = 0.755). There were 53.9% and 65.5% patients in relatively good performance status (P = 0.145), 74.8% and 81.0% patients without bone marrow involvement (P = 0.357), 53.0% and 55.2% patients with less than 2 extranodal sites involved (P = 0.791) respectively in the training and validation cohort. Meanwhile, over half of the patients presented advanced Ann Arbor stage (61.7% and 56.9%, P = 0.539) and B symptoms (64.3% and 53.4%, P = 0.166) when initially diagnosed. Nevertheless, some features, such as subtype composition, HDL-C level, treatment schedules, therapeutic responses and survival outcomes, were significantly distinct between two cohorts. Comparatively speaking, patients with angioimmunoblastic T-cell lymphoma (AITL) accounted for a higher proportion (21.7%) in the training cohort while anaplastic large cell lymphoma (ALCL) patients occupied more proportions (19.0%) in the validation cohort. The mainstream treatment therapy of the training cohort was CT alone (52.2%), however, more patients chose CT plus RT (22.4%) and CT plus HDACi (37.9%) in the validation cohort. The validation cohort achieved a higher overall response rate (ORR, 41.4% vs. 26.1%, P = 0.04) and a lower mortality (27.6% vs. 58.3%, P = 0.000). Nonetheless, the median OS (21.0 [9.0, 55.0] vs. 13.5 [5.4, 17.0], P = 0.000) and PFS (12.0 [3.5, 41.0] vs. 9.8 [3.9, 15.6], P = 0.038) of the training cohort was longer than the validation cohort. This might be explained by the limited follow-up time since that the training cohort was composed of patients from January 2006 to December 2016 and the validation cohort consisted of patients from January 2017 to June 2020.

**Table 1:** Clinical and biologic characteristics of the patients in two cohorts

Variables	Training cohort (n = 115)	Validation cohort (n = 58)	P-value
Basic results			
Age, years	54 (43.0, 65.0)	52 (45.8, 61.2)	0.611
Sex (%)			
Female	35 (30.4)	19 (32.8)	0.755
Male	80 (69.6)	39 (67.2)	
Subtype composition			
ENKTL	40 (34.8)	24 (41.4)	0.037
PTCL-NOS	32 (27.8)	14 (24.1)	
AITL	25 (21.7)	4 (6.9)	
ALCL	8 (7.0)	11 (19.0)	
EATCL	6 (5.2)	2 (3.4)	
MF/SPTCL	4 (3.5)	3 (5.2)	
ECOG score (%)			
< 2	62 (53.9)	38 (65.5)	0.145
≥ 2	53 (46.1)	20 (34.5)	
BM involvement (%)			
Absence	86 (74.8)	47 (81.0)	0.357
Presence	29 (25.2)	11 (19.0)	
Extranodal sites (%)			
< 2	61 (53.0)	32 (55.2)	0.791
≥ 2	54 (47.0)	26 (44.8)	
Ann Arbor stage (%)			
I/II	44 (38.3)	25 (43.1)	0.539
III/IV	71 (61.7)	33 (56.9)	
B symptoms (%)			
Absence	41 (35.7)	27 (46.6)	0.166
Presence	74 (64.3)	31 (53.4)	
Serological results			
LDH, U/L	243.0 (183.6, 388.0)	224.3 (178.9, 320.6)	0.433
Β2-MG, mg/L	3.0 (2.2, 3.8)	2.8 (2.2, 3.6)	0.424
TC, mmol/L	4.0 (3.2, 4.7)	4.1 (3.5, 5.1)	0.087
HDL-C, mmol/L	1.0 (0.8, 1.2)	1.1 (0.9, 1.3)	0.041
TG, mmol/L	1.3 (1.0,1.7)	1.2 (0.8,1.8)	0.375
Clinical scoring systems			
IPI (%)			
Low risk	45 (39.1)	24 (41.4)	0.819
Low-intermediate risk	13 (11.3)	9 (15.5)	
Intermediate-high risk	25 (21.8)	11 (19.0)	
High risk	32 (27.8)	14 (24.1)	
Treatment			
Therapy (%)			
CT alone	60 (52.2)	17 (29.3)	0.014
RT alone	8 (6.9)	4 (6.9)	
CT + RT	18 (15.7)	13 (22.4)	
CT + HDACi	21 (18.3)	22 (37.9)	
CT + HSCT	8 (6.9)	2 (3.5)	
Outcomes			
Therapeutic response (%)			
CR/PR	30 (26.1)	24 (41.4)	0.040
SD/PD	85 (73.9)	34 (58.6)	
Death (%)	67 (58.3)	16 (27.6)	0.000
Median OS	21.0 (9.0, 55.0)	13.5 (5.4, 17.0)	0.000
Progression and NRM (%)	78 (67.8)	26 (44.8)	0.004
Median PFS	12.0 (3.5, 41.0)	9.8 (3.9, 15.6)	0.038

*ENKTL* extranodal NK/T-cell lymphoma, *PTCL-NOS* peripheral T-cell lymphoma, not otherwise specified, *AITL* angioimmunoblastic T-cell lymphoma, *ALCL* anaplastic large cell lymphoma, *EATCL* enteropathy-associated T-cell lymphoma, *MF* mycosis fungoides, *SPTCL* subcutaneous panniculitis-like T-cell lymphoma, *ECOG* Eastern Cooperative Oncology Group, *BM* bone marrow, *LDH* lactate dehydrogenase, *β2-MG* beta-2 microglobulin, *TC* total cholesterol, *HDL-C* high-density lipoprotein cholesterol, *TG* triglycerides, *IPI* International Prognostic Index, *CT* chemotherapy, *RT* radiotherapy, *HDACi* histone deaceylase inhibitor, *HSCT* hematopoietic stem cell transplantation, *CR* complete response, *PR* partial response, *SD* stable disease, *PD* progressive disease, *OS* overall survival, *NRM* non-relapse mortality, *PFS* progression-free survival

### Association between dysregulated lipid metabolism and patient’s survival

We further investigated the prognostic significance of aberrant serum lipid metabolism. Firstly, ROC curves presented that the best cutoff values of serum TC, HDL-C and TG were 3.58, 0.95 and 1.42 mmol/L, respectively. The AUC of LDL-C was 0.578 (P = 0.154), which was not statistically significant. Thus, LDL-C could not serve as a prognostic indicator in MTCLs. Univariate Cox analysis showed that TC ≤ 3.58 mmol/L ([HR] with 95% confidence interval [CI] = 7.604 [4.507, 12.831], P = 0.000), HDL-C ≤ 0.95 mmol/L (HR [95% CI] = 2.364 [1.460, 3.828], P = 0.000) and TG** > **1.42 mmol/L (HR [95% CI] = 2.412 [1.483, 3.922], P = 0.000) were significantly related to patient’s OS (Table 2). Multivariate analysis further indicated that TC ≤ 3.58 mmol/L was an independent prognostic factor for the OS of patients (HR [95% CI] = 3.338 [1.732, 6.434], P = 0.000). The same conclusion was reached in the univariate and multivariate analysis of PFS (Additional file 1: Table S1). These results demonstrated that serum lipid metabolism level was significantly correlated with MTCL patients’ OS and PFS, in which serum TC and HDL-C level was positively correlated while TG level was negatively correlated. Notably, serum TC level could independently predict the survival outcomes of MTCL patients.

**Table 2:** Univariate and multivariate analysis of OS in the training cohort

Basic results	Univariate analysis				Multivariate analysis			
	B	SE	HR (95% CI)	P-value	B	SE	HR (95% CI)	P-value
Age, ≥ 60 vs. < 60, years	0.937	0.248	2.553 (1.570, 4.151)	**0.000**				
Sex, male vs. female	− 0.039	0.267	0.961 (0.569, 1.623)	0.883				
ECOG score, ≥ 2 vs. < 2	1.311	0.259	3.710 (2.232, 6.167)	**0.000**				
BM involvement, presence vs. absence	0.988	0.261	2.687 (1.612, 4.480)	**0.000**				
Extranodal sites, ≥ 2 vs. < 2	1.991	0.293	7.326 (4.125, 13.010)	**0.000**	0.892	0.424	2.439 (1.062, 5.602)	**0.036**
Ann Arbor stage, III/IV vs. I/II	1.209	0.296	3.350 (1.874, 5.988)	**0.000**				
B symptoms, presence vs. absence	0.446	0.264	1.563 (0.931, 2.623)	0.091				
LDH, ≥ 250 vs. < 250, U/L	1.305	0.256	3.687 (2.231, 6.090)	**0.000**				
β2-MG, ≥ 3 vs. < 3, mg/L	2.635	0.364	13.939 (6.835, 28.425)	**0.000**	1.427	0.480	4.165 (1.625, 10.674)	**0.003**
TC, ≤ 3.58 vs. > 3.58, mmol/L	2.029	0.267	7.604 (4.507, 12.831)	**0.000**	1.205	0.335	3.338 (1.732, 6.434)	**0.000**
HDL-C, ≤ 0.95 vs. > 0.95, mmol/L	0.860	0.246	2.364 (1.460, 3.828)	**0.000**				
TG, > 1.42 vs. ≤ 1.42, mmol/L	0.880	0.248	2.412 (1.483, 3.922)	**0.000**				
IPI 4/5 vs. 3 vs. 2 vs. 0/1	0.770	0.110	2.160 (1.740, 2.682)	**0.000**				

*ECOG* Eastern Cooperative Oncology Group, *BM* bone marrow, *LDH* lactate dehydrogenase, *β2-MG* beta-2 microglobulin, *TC* total cholesterol, *HDL-C* high-density lipoprotein cholesterol, *TG* triglycerides, *IPI* International Prognostic Index, *OS* overall survival, *PFS* progression-free survival, *B* regression coefficient, *SE* standard error, *HR* hazard ratio, *CI* confidence interval

Significant *P*-value < 0.05 is shown in bold

### Generation of a novel prognostic scoring system, EnBC score

After clarifying the prognostic significance of serum lipid profile, we attempted to construct a prognostic scoring system through synthetically analyzing patients’ clinicopathologic features and laboratory examinations. Thirteen variables were included in the univariate analysis and the results showed that 11 of them were significantly survival-related. The 11 variables were then brought into the multivariate regression model and the result showed that extranodal involved sites ≥ 2 (HR [95% CI] = 2.439 [1.062, 5.602], P = 0.036), β2-MG ≥ 3 mg/L (HR [95% CI] = 4.165 [1.625, 10.674], P = 0.003) and TC < 3.58 mmol/L (HR [95% CI] = 3.338 [1.732, 6.434], P = 0.000) were independent indicators for the OS of MTCL patients (Table 2). Coincidentally, these 3 factors were also independent predictors for PFS (Additional file 1: Table S1). Incorporating these three factors, we developed a novel scoring system, EnBC score. Each variable was endowed with one point in accordance with their rounded regression coefficients (B) in the multivariate analysis (Table 2, Additional file 1: Table S1). The EnBC score was calculated as a sum of points and differentiated patients into four groups: 0 point (no risk factor), 1 point (one risk factor), 2 points (two risk factors) and 3 points (three risk factors).

### Ability of EnBC score in predicting survival of MTCL patients

**Fig. 2:**
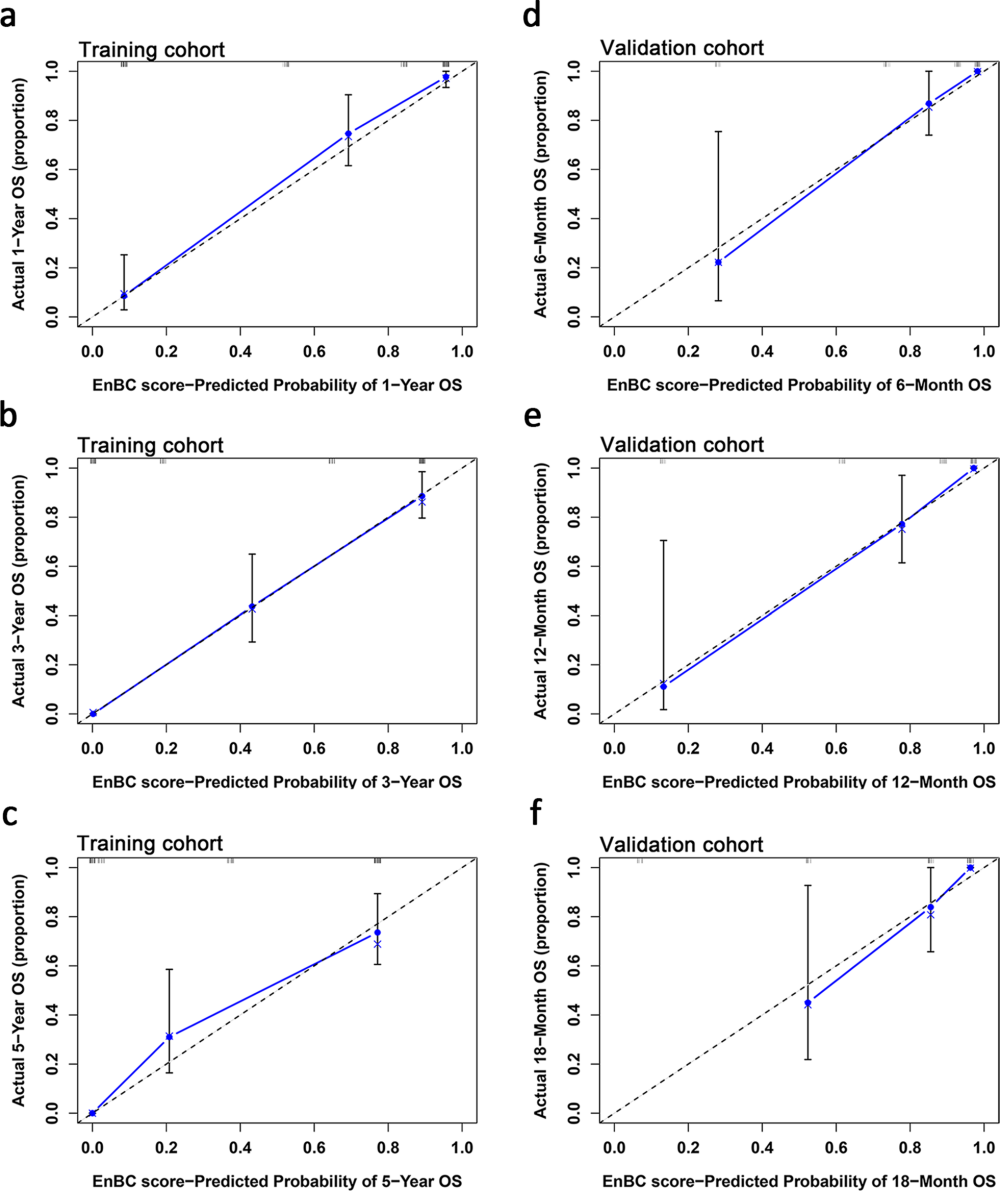
Calibration curves for the probability of OS survival in the training and validation cohort. Calibration curves reflect the consistence between the EnBC score-predicted OS and the actually-observed OS: **a** for 1-year, **b** for 3-year and **c** for 5-year prediction in the training cohort; **d** for 6-month, **e** for 12-month and **f** for 18-month prediction in validation cohort

To clarify the predictive ability of EnBC score, the discrimination and calibration were examined in the training cohort and verified in the independent validation cohort. In the training cohort, the Harrell’s C-index for OS prediction was 0.840 (95% CI 0.810–0.870) (Additional file 1: Table S2), and the calibration plot for the probability of 1-, 3-, and 5-year OS showed an optimal consistence between the prediction and actual observation (Fig. 2a–c). Similarly, in the validation cohort, the Harrell’s C-index for OS prediction was 0.882 (95% CI 0.822–0.942), with well-fitted calibration plot for the probability of 6-, 12- and 18-month OS (Fig. 2d–f).

The discrimination and calibration of EnBC score in predicting PFS were also evaluated and validated and the results were equally impressing as predicting OS (Additional file 1: Table S2 and Figure S1).

### Prognostic performance of EnBC score in survival analysis

**Fig. 3:**
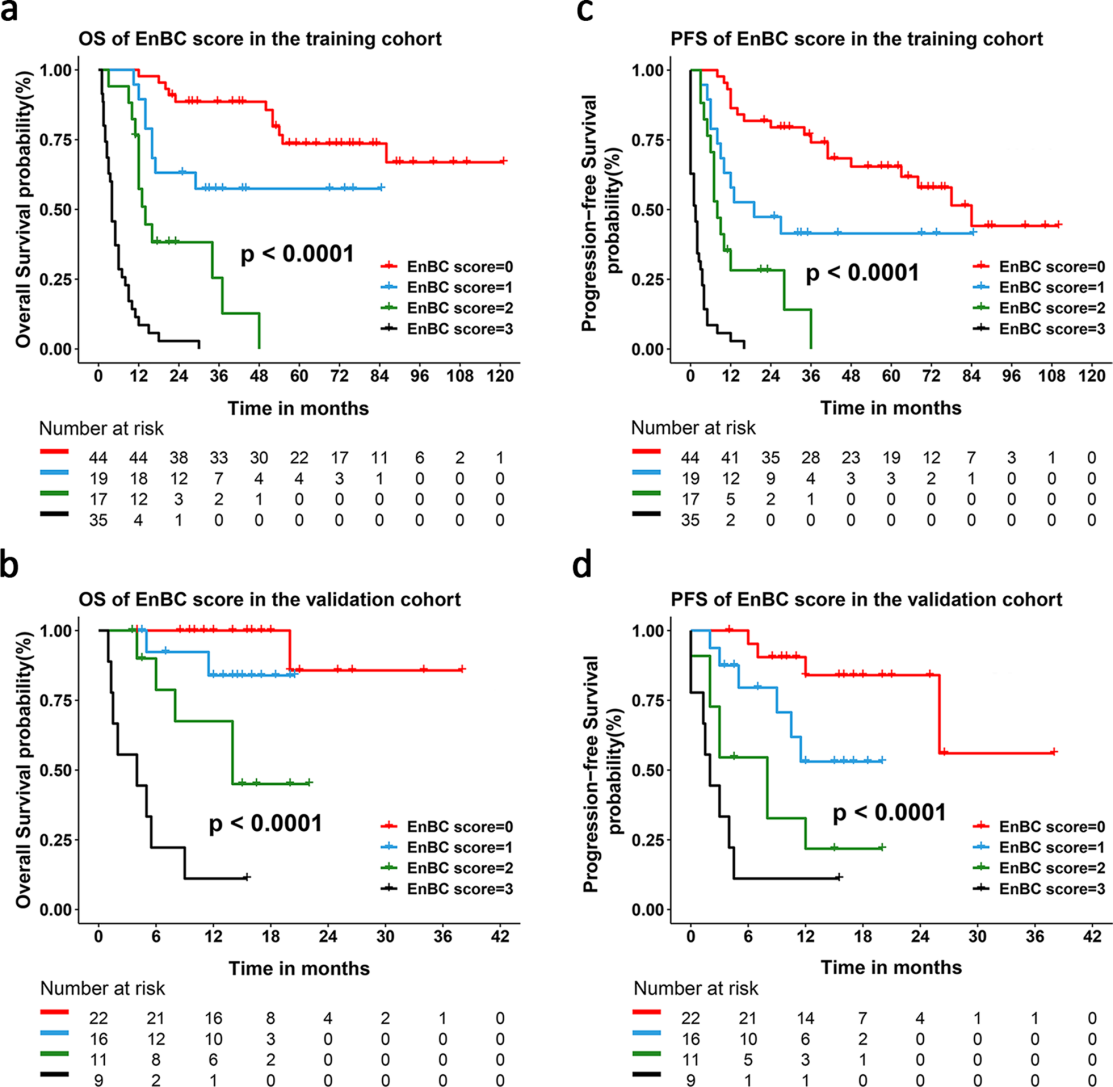
Kaplan–Meier estimated OS and PFS curves of four EnBC score grades in the training and validation cohort. Kaplan–Meier curves show the survival differences among the four grades of EnBC score: **a** for OS in the training cohort, **b** for OS in the validation cohort; **c** for PFS in the training cohort and **d** for PFS in the validation cohort

Kaplan–Meier analysis was performed to examine the survival outcomes. The median OS of the training cohort was 29 months (95% CI 2.46–55.54), and the 3- and 5-year OS rates were 47.8% and 38.6%, respectively, while median PFS was 12 months (95% CI 6.80–17.20) and the 3- and 5-year PFS rates were 36.9% and 33.1%, which were both similar to those of the validation cohort (P = 0.141 for OS, P = 0.425 for PFS, Additional file 1: Table S3 and Figure S2). The prognostic performance of EnBC score was further evaluated by dividing patients into four groups according to their points. In the training cohort, significant differences in either 3- and 5-year OS rate or the median survival time existed among the four groups (P < 0.0001, Fig. 3a). The 3-, 5-year OS rate and median OS were 88.6%, 73.6%, not available (NA) for the 0-point group, while respectively 57.4%, 57.4%, NA; 25.5%, 0.0%, 14 months (95% CI 12.0–NA) and 0.0%, 0.0%, 4 months (95% CI 2.9–5.1) for the 1-point, 2-point and 3-point groups (Additional file 1: Table S4). Equally significant differences in the 6-, 12-, and 18-month OS rate and the median OS existed among the four groups in the validation cohort (Fig. 3b and Additional file 1: Table S4). PFS differences among four groups were also confirmed in both training and validation cohort (Fig. 3c, d and Additional file 1: Table S5). Therefore, EnBC score identified four risk grades with distinct long-term survival, including low risk (EnBC score = 0), low-intermediate risk (EnBC score = 1), intermediate-high risk (EnBC score = 2) and high risk (EnBC score = 3).

### Comparison of predictive accuracy and clinical usefulness between EnBC score and IPI score

The preceding findings concluded EnBC score was a satisfactory prognostic tool for newly diagnosed MTCLs. Instinctively, we would compare it with the present scoring system, IPI score. Primarily, C-index of EnBC score for OS prediction was 0.840 and 0.882 in the training and validation cohort, significantly higher than that of IPI score, 0.749 and 0.834 (P < 0.001, P = 0.042; Additional file 1: Table S2). The similar improvement in PFS prediction was shown in Additional file 1: Table S2. These results demonstrated that EnBC score presented higher discrimination than IPI score in stratifying MTCL patients.

**Fig. 4:**
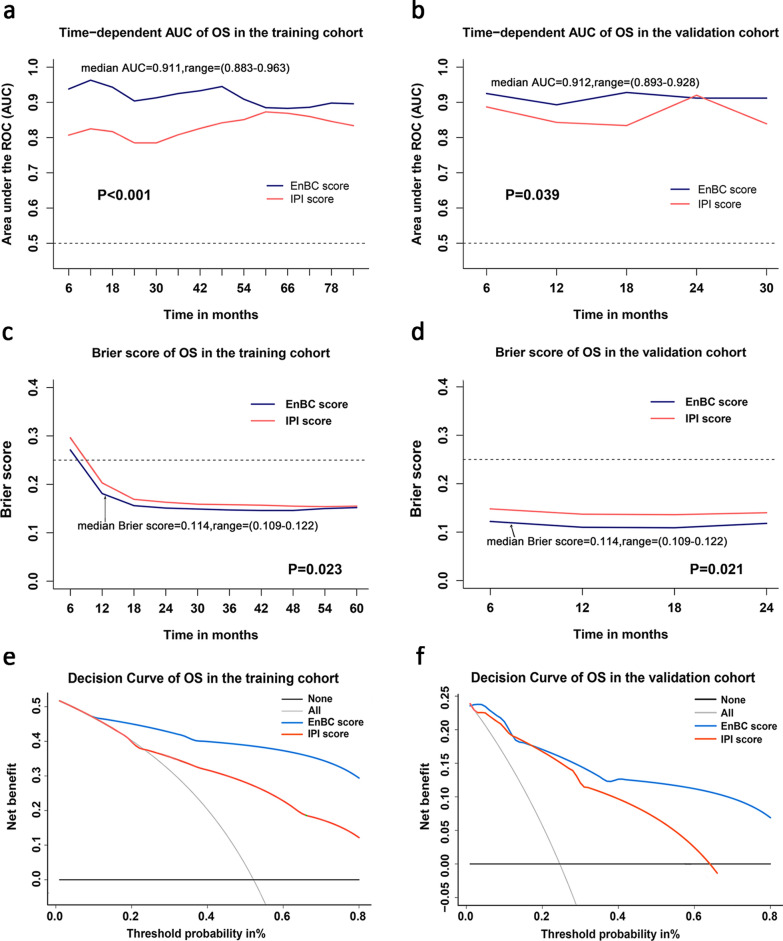
Performance of EnBC score in predicting OS in the training and validation cohort. Time-dependent AUCs measured within 84 months showed EnBC score possessed higher discrimination in OS prediction than IPI: **a** for the training cohort, **b** for the validation cohort. Brier scores calculated within 60 months presented EnBC score possessed superior calibration in OS prediction compared with IPI: **c** for the training cohort, **d** for the validation cohort. Decision curve analysis concluded EnBC score was a more useful tool for clinical decision due to the higher net benefit: **e** for the training cohort, **f** for the validation cohort

Time-dependent ROC, a more accurate discrimination parameter, was further performed to determine which scoring system behaved better in predicting OS and PFS of MTCL patients. The time-dependent AUCs of EnBC score for predicting OS within 84 months in the training cohort were 0.883–0.963, significantly higher than that of IPI score (0.785–0.873) (P < 0.001, Fig. 4a and Additional file 1: Table S6). Similar results were presented in the validation cohort. The time-dependent AUCs of EnBC score and IPI score for predicting OS within 24 months in the validation cohort were 0.893–0.928 and 0.834–0.920, respectively (P = 0.039, Fig. 4b and Additional file 1: Table S6). Besides, the time-dependent AUCs of EnBC score for predicting PFS were equally higher than those of IPI score in both training and validation cohort (P < 0.001; P = 0.114, Additional file 1: Table S6 and Figure S3). The data of time-dependent ROC further verified EnBC score was superior than IPI score in discrimination and more suitable for MTCLs.

The Brier score, quantitively reflecting the calibration of prognostic model, was calculated as well. Lower Brier score indicates higher calibration. The Brier scores of EnBC and IPI score for predicting OS within 60 months were 0.146–0.271 and 0.154–0.296, respectively, in the training cohort (P = 0.023, Fig. 4c and Additional file 1: Table S7), which indicated that EnBC score possessed higher calibration in predicting OS. The same conclusion was reached in the validation cohort (P = 0.021, Fig. 4d and Additional file 1: Table S7). As for PFS, the Brier scores of EnBC score also tended to be lower than that of IPI score (P = 0.371 in the training cohort; P = 0.268 in the validation cohort, Additional file 1: Table S7 and Figure S3). Even if the difference is not statistically significant, the curves still presented that EnBC score had higher accuracy in predicting PFS of MTCL patients.

DCA was subsequently performed to compare the clinical usefulness of EnBC score and IPI score. DCA could graphically exhibit the clinical utility of a predictive model based on a continuum of potential thresholds for risk of death (the x-axis) and the net benefit of using the model to the risk of stratifying patients relative to the assumption that no patient would be dead (the y-axis). DCA revealed that EnBC score provided superior net benefits than IPI score for OS and PFS prediction in both training and validation cohort (OS: Fig. 4e, f; PFS: Additional file 1: Figure S3). These results illuminated that stratifying MTCL patients with EnBC score could bring more benefits in the clinical practice.

## Discussion

MTCLs carry inferior prognoses in comparison with other non-Hodgkin lymphomas or Hodgkin lymphomas [15, 16]. Regrettably, MTCLs have experienced the same degree of improvement in outcome over the past 20 to 30 years [4]. At present, accurately identifying high-risk patients and then timely adjusting their treatment strategies might be an effective approach to optimize the overall survival outcomes [17].

Accurate identification and filtration of the high-risk patients rely on the advent of potent prognostic markers and scoring systems. With the advance of tools to study lipids, lipid metabolism has come into the spotlight in recent cancer researches [18]. Lipid metabolism accounts for a significant proportion in the tumor energy metabolism, even occupies a dominant position in non-glycolysis-dependent tumors, such as leukemia [19], lymphoma [19, 20] and prostate cancer [21]. Cancer stem cells highly rely upon lipid metabolism for maintaining their stemness properties [22]. Besides, altered lipid metabolism could mediate immune inhibition [23], ferroptosis escape [24] and affect the signal transduction pathway [25] to enhance tumor progression. While patient fasting status or metabolic medications might be confounders, several larger-scale lipidomic studies have provided compelling evidence for the potential of lipids to serve as diagnostic and clinically-actionable prognostic biomarkers in a range of cancers [18]. Previous studies reported that dyslipidemia was closely related to the progression of pancreatic ductal adenocarcinoma (PDAC) [26], the epithelial-to-mesenchymal transition (EMT) of prostate cancer [27] and the recurrence of early-stage, hormone receptor-positive invasive breast cancer [28]. Elevated serum cholesterol was found to serve as a potent marker of favorable outcomes in HCC [29], lung cancer [30] and esophageal cancer.

Our study for the first time demonstrated the association between the dysregulated serum lipids and the survival of MTCL patients, decreased cholesterol and increased triglycerides both portending an inferior outcome. Serum TC level was innovatively revealed its independent predictive value in MTCLs. We included TC to EnBC score according to the result of multivariate regression analysis and the stunning performance of the new model further confirmed the vital role of lipid metabolism in the progression of MTCLs.

Due to the low incidence of MTCLs, it is tough to construct the prognostic stratification systems specific for every single subtype [4]. Constrained by sample size, most MTCL subtypes lack of specific scoring systems. Only a few subtypes with relatively high morbidity possess their corresponding scoring systems, such as peripheral T cell lymphoma, not otherwise specified (PTCL-NOS) [31], ENKTL [32] and AITL [33]. Developing a universal prognostic scoring tool for MTCLs like IPI for the B-cell lymphomas might be a wiser strategy for clinical practice.

We unprecedentedly reported a specific but universal prognostic model targeting the whole MTCL patients, EnBC score. EnBC score is composed of β2-MG, extranodal involved sites and TC. The former two are commonly-recognized prognostic factors in hematological malignancies and respectively included in the existing scoring systems for chronic lymphocytic leukemia [34] and lymphomas. Contrasted with the previous systems, our study innovatively but not groundlessly included cholesterol in the scoring system. Lipids have been gradually included in the emerging prognostic indices, such as Prognostic Nutritional Index (PNI) [35], Controlling Nutritional Status (CONUT) score [36] and Naples prognostic score (NPS) [37]. In addition, lipid metabolism-related signature also showed prognostic significance for some cancers [38]. The hypothesis that tumors are driven by dysregulated metabolism largely explained the significance of metabolomics in tumor prognosis [39].

EnBC score possessed the requisite characteristics for an excellent prognostic model, discrimination, calibration, stability, simplicity and accessibility [40]. Compared with the existing scoring system, EnBC score exhibited several significant advantages for clinical practice. Firstly, EnBC score was the first specific model to predict the survival of patients with MTCLs, which was more targeted and stable than IPI score. Secondly, EnBC score integrated serological and pathological results, providing a higher predictive accuracy. Thirdly, EnBC score could effectively identify patients with a high risk of poor prognosis, thus supplying credible reference for timely adjustment of systematic treatment schedule. Finally, the predictors of EnBC score can be easily obtained, thus generating higher clinical practicability.

Nevertheless, the inevitable limitations of our study should be addressed. First, this study was carried out in a small size single-center cohort, which may lead to potential biases weakening the credibility. Second, heterogeneous therapy regimens compromised with different detailed conditions may influence the survival outcomes. Third, the EnBC score was derived from a retrospective cohort study, which had its inherent biases. Although the prospective validation cohort facilitated to increase the reliability of the study, the onset time, follow-up time and the population scale restricted the survival outcomes of the validation cohort. The prognostic efficiency of EnBC score needs to be further validated in large-scale randomized multicenter populations in the future.

## Conclusion

The study first revealed the correlation between lipid metabolism disorder and unfavorable prognosis in MTCLs. Patients with declined TC and HDL-C at the time of initial diagnosis are more likely to suffer inferior outcomes but TG reduction might be a good sign. Furthermore, we innovatively constructed a risk scoring system, EnBC score, universal for patients with any subtype of MTCLs. The novel scoring system, consisting of three simple and accessible variables, TC, β2-MG and extranodal involved sites, stratified MTCL patients into four risk grades with distinct survival. EnBC score could supply credible references for accurate risk stratification and personalized treatment due to its striking performance in discrimination, calibration and prediction of survival. The accessibility, superiority and stability assisted EnBC score to become a promising scoring tool for MTCL patients while its performance needs to be further examined in larger MTCL population.

## Supplementary Information

**Additional file 1: Table S1.** Univariate and multivariate analysis of PFS in the training cohort. **Table S2.** Harrell’s c-index of EnBC score and IPI score in predicting survival. **Table S3.** OS/PFS probability and median OS/PFS time of the training and validation cohort. **Table S4.** OS probability and median OS time of different EnBC and IPI risk grades in the training and validation cohort. **Table S5.** PFS probability and median PFS time of different EnBC and IPI risk grades in the training and validation cohort. **Table S6.** Time-dependent AUCs and 95% CI at different times of EnBC and IPI for survival prediction in the training and validation cohort. **Table S7.** Brier scores at different times of two scoring systems for survival predicting in the training and validation cohort. **Figure S1.** Calibration curves of PFS in the training and validation cohort. **Figure S2.** Kaplan–Meier estimated OS and PFS curves in the training and validation cohort. **Figure S3.** Time-dependent AUCs, Brier scores and Decision curves of PFS in the training and validation cohort.

## Data Availability

The dataset used during the study are available from the corresponding author on a reasonable request.

## Abbreviations

MTCLs: Mature T-cell lymphomas
ENKTL: Extranodal NK/T-cell lymphoma
PTCL-NOS: Peripheral T-cell lymphoma, not otherwise specified
AITL: Angioimmunoblastic T-cell lymphoma
ALCL: Anaplastic large cell lymphoma
ATLL: Adult T-cell leukemia/lymphoma
EATL: Enteropathy-associated T-cell lymphoma
MF: Mycosis fungoides
SPTCL: Subcutaneous panniculitis-like T-cell lymphoma
HCC: Hepatocellular carcinoma
PDAC: Pancreatic ductal adenocarcinoma
CLL: Chronic lymphocytic leukemia
EMT: Epithelial-to-mesenchymal transition
IPI: International Prognostic Index
PNI: Prognostic Nutritional Index
CONUT: Controlling nutritional status
NPS: Naples prognostic score
ECOG: Eastern Cooperative Oncology Group
BM: Bone marrow
LDH: Lactate dehydrogenase
β2-MG: Beta-2 microglobulin
TC: Total cholesterol
HDL-C: High-density lipoprotein cholesterol
TG: Triglycerides
LDL-C: Low-density lipoprotein cholesterol
CT: Chemotherapy
RT: Radiotherapy
CRT: Chemoradiotherapy
IMRT: Intensity-modulated radiotherapy
HDACi: Histone deaceylase inhibitor
HSCT: Hematopoietic stem cell transplantation
CR: Complete response
PR: Partial response
SD: Stable disease
PD: Progressive disease
ORR: Overall response rate
OS: Overall survival
NRM: Non-relapse mortality
PFS: Progression-free survival
SDs: Standard deviations
ROC: Receiver operating characteristic
DCA: Decision curve analysis
AUCs: Areas under ROC curve
HR: Hazard ratio
CI: Confidence interval
NA: Not available
