# Supplementary material for: Derivation and validation of a lipid-covered prognostic model for mature T-cell lymphomas
Source: Cancer Cell Int. 2021 Jul 5;21:348. doi: 10.1186/s12935-021-02042-3 (PMC8256497; doi:10.1186/s12935-021-02042-3)

Additional file 1

Table S1. Univariate and multivariate analysis of PFS in the training cohort.

	Univariate analysis				Multivariate analysis			
Basic results	B	SE	HR (95%CI)	P-value	B	SE	HR (95%CI)	P-value
Age, ≥60 vs. < 60, years	0.771	0.230	2.161(1.376,3.395)	0.001				
Sex, male vs. female	0.039	0.249	1.040(0.639,1.693)	0.875				
ECOG score, ≥2 vs. < 2	1.259	0.238	3.521(2.211,5.609)	0.000				
BM involvement, presence vs. absence	0.865	0.250	2.375(1.455,3.876)	0.001				
Extranodal sites, ≥2 vs. < 2	1.740	0.255	5.697(3.455,9.396)	0.000	1.068	0.384	2.911 (1.373,6.173)	0.005
Ann Arbor Stage, III/IV vs. I/II	0.906	0.254	2.476(1.503,4.076)	0.000				
B symptoms, presence vs. absence	0.417	0.245	1.518(0.940,2.452)	0.088				
LDH, ≥250 vs. < 250, U/L	0.970	0.232	2.637(1.673,4.157)	0.000				
β2-MG, ≥3 vs. < 3, mg/L	2.122	0.293	8.350(4.698,14.843)	0.000	1.080	0.411	2.943(1.316,6.585)	0.009
TC, ≤3.58 vs. > 3.58, mmol/L	1.760	0.240	5.811(3.629,9.305)	0.000	0.920	0.304	2.508(1.383,4.549)	0.002
HDL-C, ≤0.95 vs. > 0.95, mmol/L	0.946	0.230	2.576(1.640,4.045)	0.000				
TG, > 1.42 vs. ≤1.42, mmol/L	0.869	0.230	2.384(1.518,3.744)	0.000				
IPI 4/5 vs.3 vs.2 vs.0/1	0.659	0.099	0.932(1.591,2.346)	0.000				

Abbreviations: ECOG: Eastern Cooperative Oncology Group; BM: bone marrow; LDH: lactate dehydrogenase; β2-MG: beta-2 microglobulin; TC, total cholesterol; HDL-C, high-density lipoprotein cholesterol; TG, triglycerides; IPI: international prognostic index; OS: overall survival; PFS: progression-free survival; B, coefficient; SE, standard error; HR, Hazard ratio; CI, confidence interval.

Table S2. Harrell's c-index of EnBC score and IPI score in predicting survival.

	Cohort	Scoring systems	Harrell's c-index	Up	Down	P (compared)
OS	Training Cohort	EnBC score	0.840	0.810	0.870	
		IPI score	0.749	0.698	0.801	<0.001
	Validation Cohort	EnBC score	0.882	0.822	0.942	
		IPI score	0.834	0.763	0.906	0.042
PFS	Training Cohort	EnBC score	0.823	0.792	0.853	
		IPI score	0.727	0.675	0.778	<0.001
	Validation Cohort	EnBC score	0.804	0.729	0.880	
		IPI score	0.765	0.677	0.853	0.045

Abbreviations: OS: overall survival; PFS: progression-free survival; IPI: international prognostic index.

Figure S1. Calibration curves of PFS in the training and validation cohort.

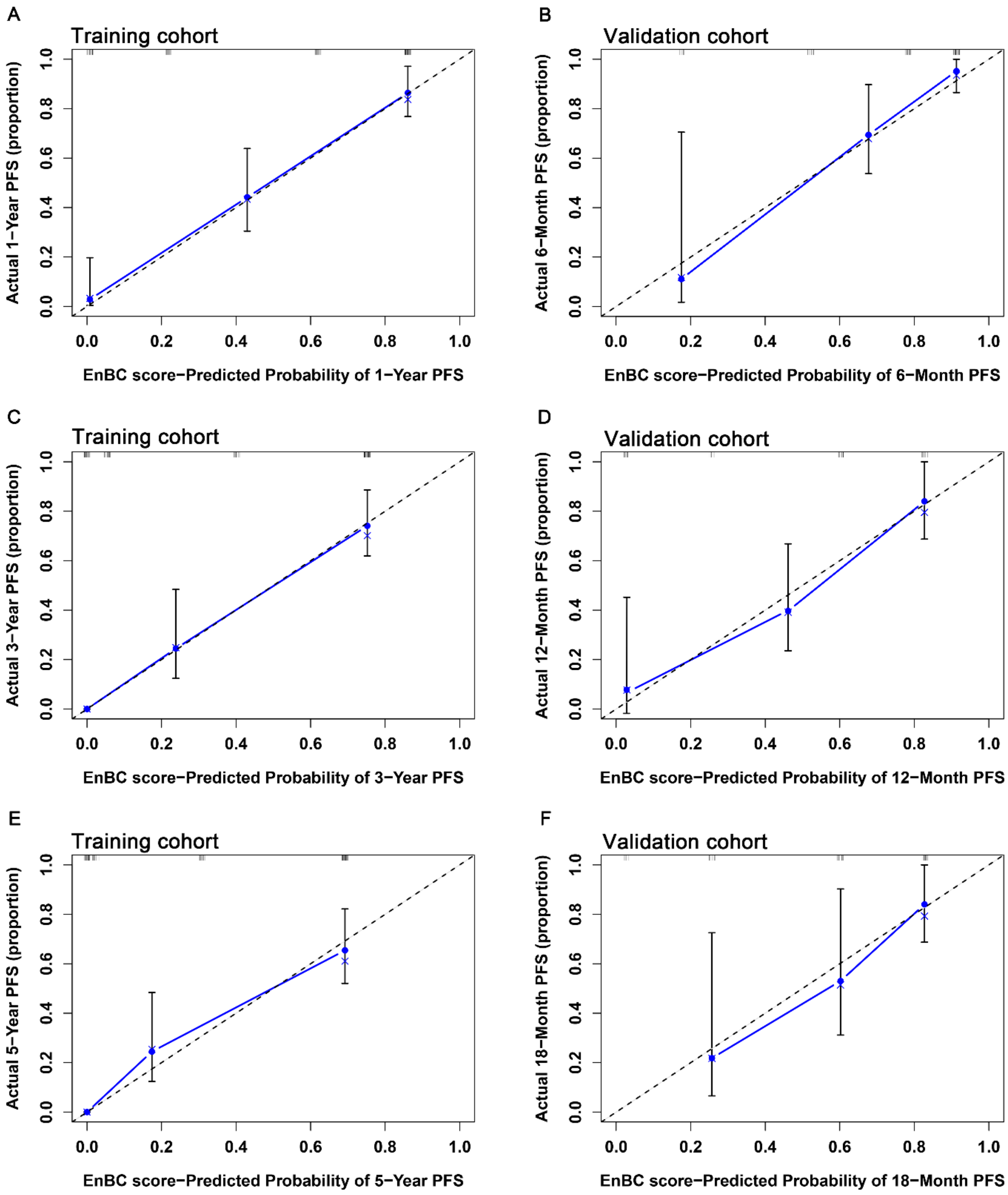

Table S3. OS/PFS probability and median OS/PFS time of the training and validation cohort.

	Training Cohort (n=115)		Validation Cohort (n=58)		p value
	Month rate and median survival time		Month rate and median survival time		
OS	12-month Probability	63.4%	6-month Probability	81.7%	-
	36-month Probability	47.8%	12-month Probability	75.4%	-
	60-month Probability	38.6%	18-month Probability	70.2%	-
	Median survival time (months)	29.00 (2.46-55.54)	Median survival time (months)	NA	0.141
PFS	12-month Probability	47.7%	6-month Probability	70.1%	-
	36-month Probability	36.9%	12-month Probability	52.6%	-
	60-month Probability	33.1%	18-month Probability	52.6%	-
	Median survival time (months)	12.00 (6.80-17.20)	Median survival time (months)	26.00 (6.80-45.20)	0.425

Abbreviations: OS: overall survival; PFS: progression-free survival; NA: Not available.

Figure S2. Kaplan-Meier estimated OS and PFS curves in the training and validation cohort.

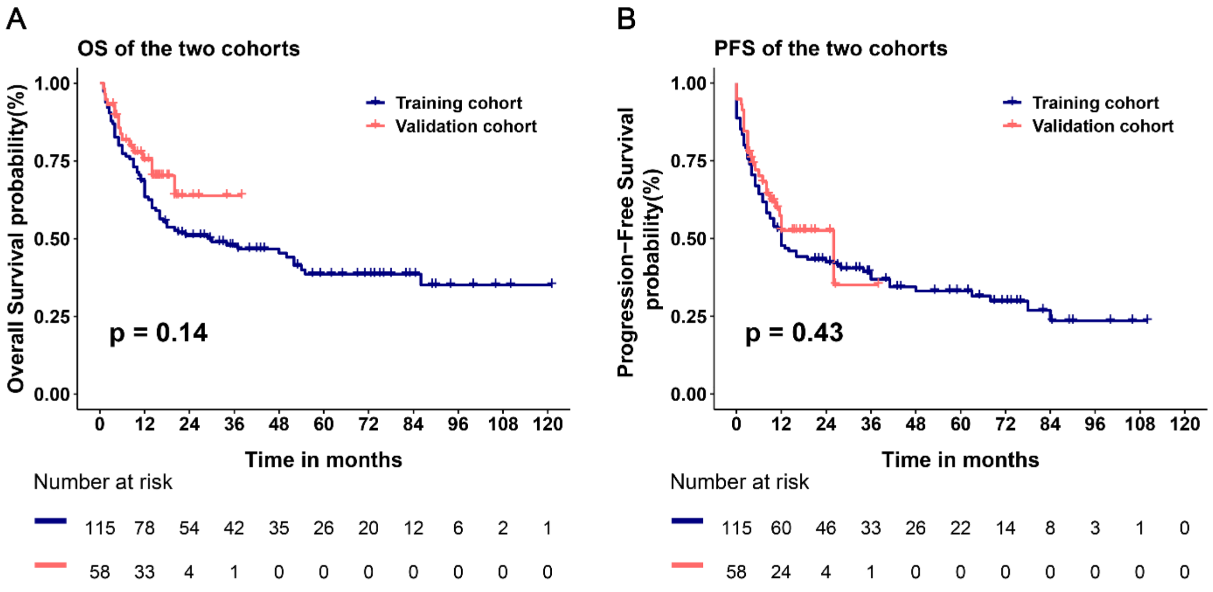

Table S4. OS probability and median OS time of different EnBC and IPI risk grades in the training and validation cohort.

Current Scoring systems	Risk Grades	Training Cohort (n=115)		Validation Cohort (n=58)		P value
		Month rate and median survival time (months)		Month rate and median survival time (months)		
EnBC Score	Low	12-month probability	97.7%	6-month probability	100.0%	
		36-month probability	88.6%	12-month probability	100.0%	
		60-month probability	73.6%	18-month probability	100.0%	
		Median survival time	NA	Median survival time	NA	0.918
	Low-intermediate	12-month probability	89.5%	6-month probability	92.3%	
		36-month probability	57.4%	12-month probability	83.9%	
		60-month probability	57.4%	18-month probability	83.9%	
		Median survival time	NA	Median survival time	NA	0.520
	Intermediate-high	12-month probability	57.4%	6-month probability	78.8%	
		36-month probability	25.5%	12-month probability	67.5%	
		60-month probability	0.0%	18-month probability	45.0%	
		Median survival time	14.0 (12.0-NA)	Median survival time	14.0 (5.3-22.7)	0.884
	High	12-month probability	8.6%	6-month probability	22.2%	
		36-month probability	0.0%	12-month probability	11.1%	
		60-month probability	0.0%	18-month probability	NA	
		Median survival time	4.0 (2.9-5.1)	Median survival time	4.0 (0.0-9.8)	0.861
IPI Score	Low	12-month probability	91.1%	6-month probability	100.0%	
		36-month probability	77.0%	12-month probability	100.0%	
		60-month probability	73.7%	18-month probability	100.0%	
		Median survival time	94.0 (80.3-107.7)	Median survival time	NA	0.097
	Low-	12-month probability	84.6%	6-month probability	100.0%	

	intermediate	36-month probability	61.5%	12-month probability	85.7%	
		60-month probability	44.0%	18-month probability	71.4%	
		Median survival time	52.0 (1.1-102.9)	Median survival time	NA	0.999
	Intermediate-high	12-month probability	48.0%	6-month probability	72.7%	
		36-month probability	36.0%	12-month probability	63.6%	
		60-month probability	0.0%	18-month probability	63.6%	
		Median survival time	12.0 (7.1-16.9)	Median survival time	20.0 (NA-NA)	0.391
	High	12-month probability	28.1%	6-month probability	44.9%	
		36-month probability	11.3%	12-month probability	35.9%	
		60-month probability	3.8%	18-month probability	26.9%	
		Median survival time	6.0 (0.0-12.9)	Median survival time	6.0 (2.9-9.1)	0.790

Abbreviations: OS: overall survival; IPI: international prognostic index; NA: Not available.

Table S5. PFS probability and median PFS time of different EnBC and IPI risk grades in the training and validation cohort.

Current Scoring systems	Grade	Training Cohort (n=115)		Validation Cohort (n=58)		P value
		Month rate and median survival time (months)		Month rate and median survival time (months)		
EnBC Score	Low	12-month probability	86.4%	6-month probability	95.2%	
		36-month probability	74.1%	12-month probability	84.0%	
		60-month probability	65.4%	18-month probability	84.0%	
		Median survival time	84.0 (59.7-108.3)	Median survival time	NA	0.587
	Low-intermediate	12-month probability	57.9%	6-month probability	79.5%	
		36-month probability	41.4%	12-month probability	53.0%	
		60-month probability	41.4%	18-month probability	53.0%	
		Median survival time	19.0 (0.0-39.5)	Median survival time	NA	0.950
	Intermediate-high	12-month probability	28.2%	6-month probability	54.5%	
		36-month probability	14.1%	12-month probability	21.8%	
		60-month probability	0.0%	18-month probability	21.8%	
		Median survival time	8.0 (5.6-10.4)	Median survival time	8.0 (3.6-12.4)	0.457
	High	12-month probability	2.9%	6-month probability	11.1%	
		36-month probability	0.0%	12-month probability	11.1%	
		60-month probability	0.0%	18-month probability	NA	
		Median survival time	1.5 (0.3-2.7)	Median survival time	2.0 (1.5-3.5)	0.464
IPI Score	Low	12-month probability	75.4%	6-month probability	95.2%	
		36-month probability	66.1%	12-month probability	90.5%	
		60-month probability	62.9%	18-month probability	90.5%	
		Median survival time	84.0 (NA-NA)	Median survival time	26.0 (NA-NA)	0.334

		12-month probability	69.2%	6-month probability	77.8%	
	Low-intermediate	36-month probability	52.7%	12-month probability	15.6%	
		60-month probability	44.0%	18-month probability	15.6%	
		Median survival time	41.0 (0.0-85.5)	Median survival time	8.0 (4.0-12.0)	0.021
		12-month probability	28.0%	6-month probability	45.5%	
	Intermediate-high	36-month probability	16.0%	12-month probability	36.4%	
		60-month probability	0.0%	18-month probability	36.4%	
		Median survival time	5.0 (3.5-6.5)	Median survival time	5.0 (0.0-13.1)	0.596
		12-month probability	15.6%	6-month probability	42.9%	
	High	36-month probability	4.2%	12-month probability	25.7%	
		60-month probability	4.2%	18-month probability	25.7%	
		Median survival time	2.0 (0.0-4.8)	Median survival time	3.0 (0.0-7.6)	0.403

Abbreviations: PFS: progression-free survival; IPI: international prognostic index; NA: Not available.

Table S6. Time-dependent AUCs and 95%CI at different times of EnBC and IPI for survival prediction in the training and validation cohort.

Cohort	Month rate	OS		PFS	
		EnBC score	IPI score	EnBC score	IPI score
Training Cohort (n=115)	6-month	0.938(0.901-0.974)	0.807(0.724-0.891)	0.954(0.920-0.989)	0.815(0.736-0.894)
	12-month	0.963(0.932-0.994)	0.825(0.746-0.905)	0.922(0.872-0.972)	0.806(0.725-0.887)
	18-month	0.943(0.903-0.983)	0.817(0.740-0.895)	0.887(0.829-0.944)	0.776(0.694-0.859)
	24-month	0.904(0.847-0.960)	0.785(0.702-0.869)	0.897(0.842-0.951)	0.781(0.697-0.865)
	30-month	0.913(0.860-0.966)	0.785(0.699-0.870)	0.897(0.843-0.951)	0.783(0.700-0.867)
	36-month	0.925(0.877-0.974)	0.808(0.725-0.891)	0.905(0.854-0.956)	0.817(0.738-0.896)
	42-month	0.933(0.888-0.979)	0.826(0.746-0.906)	0.878(0.821-0.936)	0.811(0.730-0.892)
	48-month	0.945(0.904-0.986)	0.842(0.762-0.921)	0.882(0.826-0.939)	0.830(0.750-0.909)
	54-month	0.909(0.854-0.964)	0.851(0.769-0.934)	0.873(0.814-0.931)	0.830(0.749-0.911)
	60-month	0.885(0.824-0.947)	0.873(0.780-0.946)	0.871(0.811-0.931)	0.838(0.757-0.920)
	66-month	0.883(0.821-0.946)	0.869(0.793-0.945)	0.857(0.792-0.921)	0.820(0.729-0.911)
	72-month	0.886(0.824-0.948)	0.860(0.774-0.945)	0.849(0.781-0.917)	0.811(0.701-0.920)
	78-month	0.898(0.840-0.955)	0.846(0.738-0.953)	0.855(0.786-0.924)	0.847(0.771-0.922)
	84-month	0.896(0.836-0.955)	0.834(0.714-0.955)	0.832(0.749-0.915)	0.842(0.760-0.924)
Validation Cohort (n=58)	6-month	0.925(0.840-1.000)	0.887(0.805-0.970)	0.904(0.828-0.980)	0.842(0.743-0.941)
	12-month	0.893(0.798-0.988)	0.843(0.734-0.952)	0.813(0.685-0.940)	0.729(0.574-0.884)
	18-month	0.928(0.844-1.000)	0.834(0.686-0.981)	0.837(0.701-0.973)	0.760(0.570-0.950)
	24-month	0.912(0.770-1.053)	0.920(0.775-1.064)	0.934(0.864-1.004)	0.851(0.655-1.048)

Abbreviations: AUCs: Areas under the ROC curve (ROC: Receiver operating characteristic curve); CI: Confidence interval; OS, overall survival; PFS: progression-free survival; IPI: international prognostic index.

Table S7. Brier scores at different times of two scoring systems for survival predicting in the training and validation cohort.

Cohort	Month rate	OS		PFS	
		EnBC score	IPI score	EnBC score	IPI score
Training Cohort (n=115)	6-month	0.271	0.296	0.250	0.261
	12-month	0.181	0.203	0.189	0.183
	18-month	0.156	0.169	0.181	0.174
	24-month	0.151	0.163	0.176	0.170
	30-month	0.149	0.159	0.172	0.166
	36-month	0.147	0.158	0.166	0.161
	42-month	0.146	0.157	0.164	0.160
	48-month	0.146	0.155	0.164	0.159
	54-month	0.150	0.154		
	60-month	0.152	0.155		
Validation Cohort (n=58)	6-month	0.122	0.148	0.197	0.218
	12-month	0.110	0.137	0.175	0.193
	18-month	0.109	0.136	0.174	0.193
	24-month	0.118	0.140		

Abbreviations: OS, overall survival; PFS, progression-free survival; IPI: international prognostic index.

Figure S3. Time-dependent AUCs, Brier scores and Decision curves of PFS in the training and validation cohort.

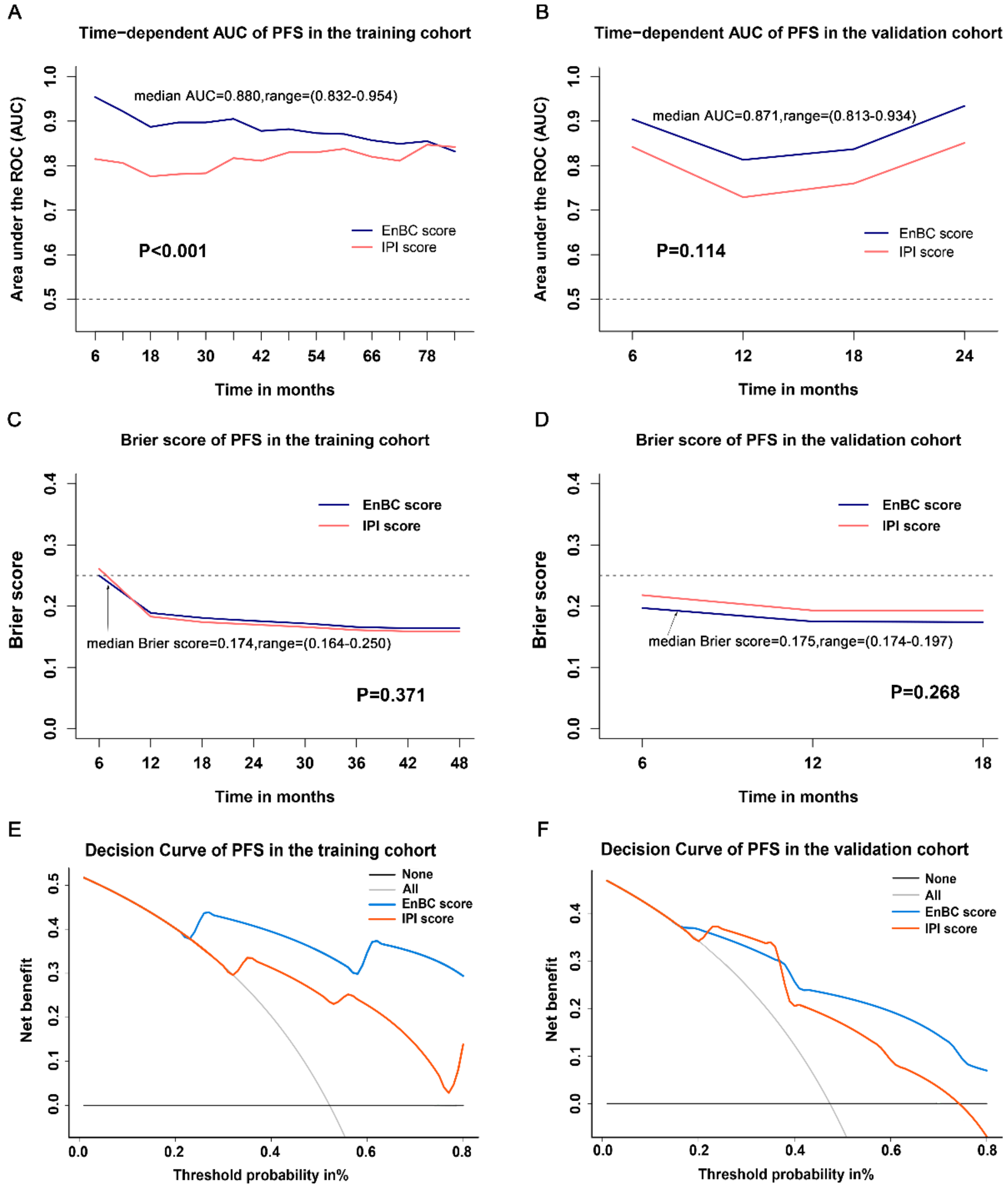

Supplement: Supplementary file 1 — Additional file 1: Table S1. Univariate and multivariate analysis of PFS in the training cohort. Table S2. Harrell’s c-index of EnBC score and IPI score in predicting survival. Table S3. OS/PFS probability and median OS/PFS time of the training and validation cohort. Table S4. OS probability and median OS time of different EnBC and IPI risk grades in the training and validation cohort. Table S5. PFS probability and median PFS time of different EnBC and IPI risk grades in the training and validation cohort. Table S6. Time-dependent AUCs and 95% CI at different times of EnBC and IPI for survival prediction in the training and validation cohort. Table S7. Brier scores at different times of two scoring systems for survival predicting in the training and validation cohort. Figure S1. Calibration curves of PFS in the training and validation cohort. Figure S2. Kaplan–Meier estimated OS and PFS curves in the training and validation cohort. Figure S3. Time-dependent AUCs, Brier scores and Decision curves of PFS in the training and validation cohort. [file 12935_2021_2042_MOESM1_ESM.pdf]
